# Imaging Severity COVID-19 Assessment in Vaccinated and Unvaccinated Patients: Comparison of the Different Variants in a High Volume Italian Reference Center

**DOI:** 10.3390/jpm12060955

**Published:** 2022-06-10

**Authors:** Vincenza Granata, Roberta Fusco, Alberta Villanacci, Simona Magliocchetti, Fabrizio Urraro, Nardi Tetaj, Luisa Marchioni, Fabrizio Albarello, Paolo Campioni, Massimo Cristofaro, Federica Di Stefano, Nicoletta Fusco, Ada Petrone, Vincenzo Schininà, Francesca Grassi, Enrico Girardi, Stefania Ianniello

**Affiliations:** 1 Division of Radiology, Istituto Nazionale Tumori IRCCS Fondazione Pascale—IRCCS di Napoli, 80131 Naples, Italy; v.granata@istitutotumori.na.it; 2 Medical Oncology Division, Igea SpA, 80013 Napoli, Italy; 3 Diagnostic Imaging of Infectious Diseases, National Institute for Infectious Diseases Lazzaro Spallanzani IRCCS, 00149 Rome, Italy; alberta.villanacci@inmi.it (A.V.); fabrizio.albarello@inmi.it (F.A.); paolo.campioni@inmi.it (P.C.); massimo.cristofaro@inmi.it (M.C.); federica.distefano@inmi.it (F.D.S.); nicoletta.fusco@inmi.it (N.F.); ada.petrone@inmi.it (A.P.); vincenzo.schinina@inmi.it (V.S.); stefania.ianniello@inmi.it (S.I.); 4 Division of Radiology, Università degli Studi della Campania Luigi Vanvitelli, 80128 Naples, Italy; simona.magliocchetti@unicampania.it (S.M.); fabrizio.urraro@unicampania.it (F.U.); francescagrassi1996@gmail.com (F.G.); 5 Intensive Care Unit, National Institute for Infectious Diseases Lazzaro Spallanzani IRCCS, 00149 Rome, Italy; nardi.tetaj@inmi.it (N.T.); luisa.marchioni@inmi.it (L.M.); 6 Italian Society of Medical and Interventional Radiology (SIRM), SIRM Foundation, Via Della Signora 2, 20122 Milan, Italy; 7 Department of Epidemiology and Research, National Institute for Infectious Diseases Lazzaro Spallanzani IRCCS, 00149 Rome, Italy; enrico.girardi@inmi.it

**Keywords:** COVID-19, vaccination, Computed Tomography

## Abstract

Purpose: To analyze the vaccine effect by comparing five groups: unvaccinated patients with Alpha variant, unvaccinated patients with Delta variant, vaccinated patients with Delta variant, unvaccinated patients with Omicron variant, and vaccinated patients with Omicron variant, assessing the “gravity” of COVID-19 pulmonary involvement, based on CT findings in critically ill patients admitted to Intensive Care Unit (ICU). Methods: Patients were selected by ICU database considering the period from December 2021 to 23 March 2022, according to the following inclusion criteria: patients with proven Omicron variant COVID-19 infection with known COVID-19 vaccination with at least two doses and with chest Computed Tomography (CT) study during ICU hospitalization. Wee also evaluated the ICU database considering the period from March 2020 to December 2021, to select unvaccinated consecutive patients with Alpha variant, subjected to CT study, consecutive unvaccinated and vaccinated patients with Delta variant, subjected to CT study, and, consecutive unvaccinated patients with Omicron variant, subjected to CT study. CT images were evaluated qualitatively using a severity score scale of 5 levels (none involvement, mild: ≤25% of involvement, moderate: 26–50% of involvement, severe: 51–75% of involvement, and critical involvement: 76–100%) and quantitatively, using the Philips IntelliSpace Portal clinical application CT COPD computer tool. For each patient the lung volumetry was performed identifying the percentage value of aerated residual lung volume. Non-parametric tests for continuous and categorical variables were performed to assess statistically significant differences among groups. Results: The patient study group was composed of 13 vaccinated patients affected by the Omicron variant (Omicron V). As control groups we identified: 20 unvaccinated patients with Alpha variant (Alpha NV); 20 unvaccinated patients with Delta variant (Delta NV); 18 vaccinated patients with Delta variant (Delta V); and 20 unvaccinated patients affected by the Omicron variant (Omicron NV). No differences between the groups under examination were found (*p* value > 0.05 at Chi square test) in terms of risk factors (age, cardiovascular diseases, diabetes, immunosuppression, chronic kidney, cardiac, pulmonary, neurologic, and liver disease, etc.). A different median value of aerated residual lung volume was observed in the Delta variant groups: median value of aerated residual lung volume was 46.70% in unvaccinated patients compared to 67.10% in vaccinated patients. In addition, in patients with Delta variant every other extracted volume by automatic tool showed a statistically significant difference between vaccinated and unvaccinated group. Statistically significant differences were observed for each extracted volume by automatic tool between unvaccinated patients affected by Alpha variant and vaccinated patients affected by Delta variant of COVID-19. Good statistically significant correlations among volumes extracted by automatic tool for each lung lobe and overall radiological severity score were obtained (ICC range 0.71–0.86). GGO was the main sign of COVID-19 lesions on CT images found in 87 of the 91 (95.6%) patients. No statistically significant differences were observed in CT findings (ground glass opacities (GGO), consolidation or crazy paving sign) among patient groups. Conclusion: In our study, we showed that in critically ill patients no difference were observed in terms of severity of disease or exitus, between unvaccinated and vaccinated patients. The only statistically significant differences were observed, with regard to the severity of COVID-19 pulmonary parenchymal involvement, between unvaccinated patients affected by Alpha variant and vaccinated patients affected by Delta variant, and between unvaccinated patients with Delta variant and vaccinated patients with Delta variant.

## 1. Introduction

Over two years after the first described SARS-CoV-2 patient, the COVID-19 pandemic is still ongoing, with many countries undergoing new infection waves [1,2,3,4,5,6,7,8,9,10,11,12]. Extensive vaccination promotion is underway all over the world, although with extremely variable levels of population coverage [1,13,14,15,16,17,18,19,20,21,22,23,24]. In addition, the pandemic perseveres with the appearance of new variants that could compromise diagnostic tests and vaccine efficacy. Developing evidence has demonstrated that these variants are able to evade the action of neutralizing antibodies [25,26,27,28,29,30,31,32,33,34,35,36,37,38,39,40,41,42,43]. Evidence of declining vaccine immunity over time has also arisen: following the second dose, there is a substantial decline in efficacy against symptomatic infection; from a peak of ~90% in the weeks immediately following to a much lower 50–80% six months after vaccination [44,45,46,47,48]. Consequently, several nations are proposing booster vaccinations. Data from these countries have proven the benefit of a booster dose in reducing symptomatic infection and offering a significant decrease in critical outcomes [49,50,51,52,53,54]. Moreover, the protection level offered by previous SARS-CoV-2 infection, both in terms of infection and disease severity and, therefore, of outcome, is still unclear [55,56,57,58,59,60,61]. In this scenario, the main essential element leading to the evolution of SARS-CoV-2 infection is the interaction with the host’s immune system. However, there is a need to understand how the new variants can lead to severe forms of the disease, as well as how the time elapsed since vaccination can impact the outcome. An assessment of disease severity requires tools that can objectify the data to reduce the variability between patients due to qualitative evaluation. As to the “gravity assessment” of COVID-19 infection and evaluation of pulmonary parenchymal involvement, several scores have been proposed [62,63]. The main goal of these tools is to establish a well-defined strategy for evaluation of the airways and lungs of COVID-19 positive patients from Computed Tomography (CT) scans, including detected abnormalities [64,65,66,67,68,69,70,71,72,73,74]. Their identification and the volumetric quantification may allow an easier classification in terms of gravity, extent and progression of the disease. Moreover, this may provide a high-impact tool to enhance awareness of the severity of COVID-19 pneumonia [75,76,77,78,79,80,81,82,83,84,85,86,87,88,89,90].

In this retrospective cohort study, we aim to analyze the vaccine effect by comparing five groups: (a) unvaccinated patients with Alpha variant; (b) unvaccinated patients with Delta variant; (c) vaccinated patients with Delta variant; (d) unvaccinated patients with Omicron variant; and (e) vaccinated patients with Omicron variant, assessing the “gravity” of COVID-19 pulmonary involvement, based on CT findings in critically ill patients admitted to Intensive Care Unit (ICU).

## 2. Materials and Methods

### 2.1. Patient Characteristics

The study was conducted according to the guidelines of the Declaration of Helsinki and approved by the Institutional Ethics Committee of IRCCS L. Spallanzani. Data acquisition and analysis were performed in compliance with protocols approved by the Ethical Committee of the National Institute for Infectious Diseases IRCCS Lazzaro Spallanzani, Rome, Italy (ethical approval number 164, 26 June 2020). The Local Ethical Committee board renounced patient informed consent, considering the ongoing epidemic emergency.

Patients were selected from the Intensive Care Unit (ICU) database considering the period from December 2021 to 23 March 2022, having COVID-19 infection variant sequencing, according to the following inclusion criteria: (1) patients with proven Omicron variant COVID-19 infection; (2) patients with known COVID-19 vaccination with at least two doses; (3) patients with chest CT study during ICU hospitalization. The exclusion criteria were: (1) no CT study, (2) patients with no data on COVID-19 vaccination status.

We also evaluated the ICU database considering the period from March 2020 to December 2021, to select unvaccinated consecutive patients with Alpha variant, subjected to CT study; consecutive unvaccinated patients with Delta variant, subjected to CT study; consecutive vaccinated patients with Delta variant, subjected to CT study; consecutive unvaccinated patients with Omicron variant, subjected to CT study.

### 2.2. CT Technique

Chest CT scan was performed with 128 slices using Incisive Philips CT scanners (Amsterdam, The Netherlands). CT examinations were performed with the patient in the supine position in breath-hold, and inspiration using a standard dose protocol, without contrast intravenous injection. The scanning range was from the apex to the base of the lungs. The tube voltage and the current tube were 120 kV and 100–200 mA (and if applicable, using *z*-axis tube current modulation), respectively. All data were reconstructed with a 0.6–1.0 mm increment. The matrix was 512 mm × 512 mm. Images were reconstructed using a sharp reconstruction kernel for parenchyma evaluation and hard reconstruction kernel for other lung evaluation. All data were reconstructed with a 0.6–1.0 mm increment. Multiplanar reconstruction (MPR) was also obtained. 

### 2.3. CT Post Processing

DICOM data were transferred into a PACS workstation and CT images were evaluated using the Philips IntelliSpace Portal clinical application CT COPD (Philips Eindihoven, The Netherlands) computer tool.

**Figure 1:**
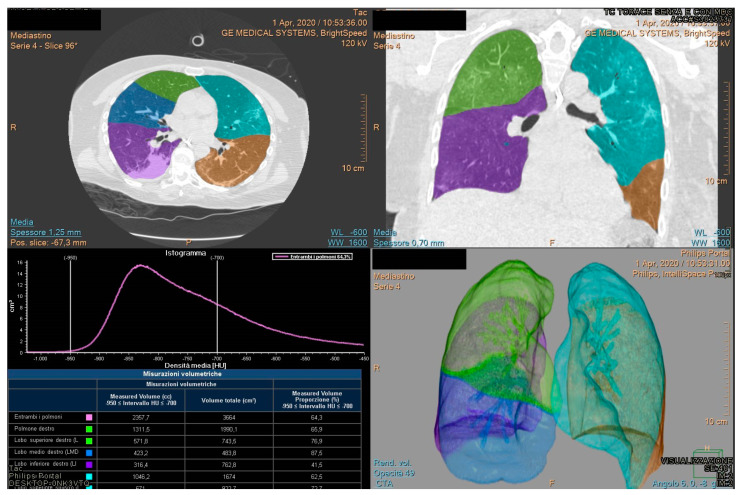
Example 1 of Quantitative assessment of COVID-19 pulmonary parenchymal involvement by automatic tool.

**Figure 2:**
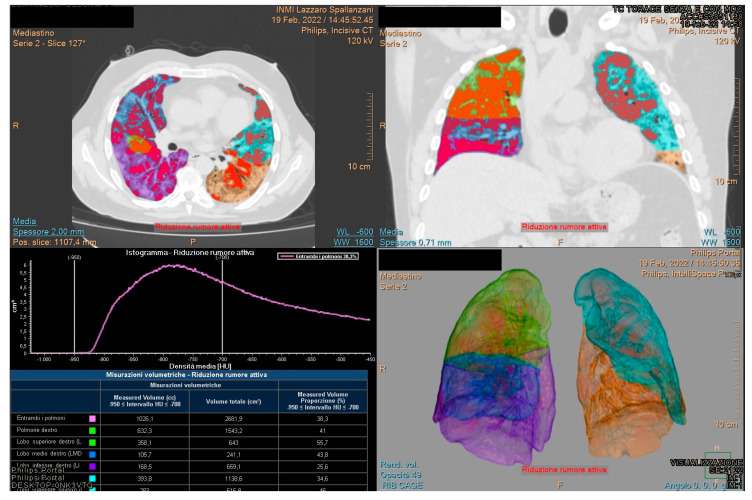
Example 2 of Quantitative assessment of COVID-19 pulmonary parenchymal involvement by automatic tool.

Philips IntelliSpace Portal clinical application CT COPD software is a CE-marked medical device designed to quantify pulmonary emphysema in patients with chronic obstructive pulmonary disease. The tool provides segmentation of the lungs and of the airway tree. Moreover, the tool helps visualize and quantify the destructive process of diffuse lung disease (e.g., emphysema), providing a guided workflow for airway analysis, reviewing and measuring airway lumen, and assessing trapped air. Compared to others tools, it allows assessment consolidation. For each patient the lung volumetry was performed identifying the percentage value of aerated residual lung volume, and for each lung lobe: right upper lobe volume, right lower lobe volume, medium lobe volume, left upper lobe volume, left lower lobe volume (Figure 1 and Figure 2).

Disease severity was assessed by considering the percentage of aerated residual lung volume: patients with lower aerated residual lung volume were considered more compromised.

### 2.4. Radiologists’ Analysis

Radiologists attributed for each lung lobe (right upper and lower lobe, medium lobe, left upper and lower lobe) a severity score using a scale of 5 levels (no involvement, mild: ≤25% of involvement, moderate: 26–50% of involvement, severe: 51–75% of involvement and critic involvement: 76–100%) as reported in Li et al. [91]. Moreover, an overall radiological severity score was obtained summing the scores for each lung lobe and then considering a low severity ≤ 5, mild severity 6–10, moderate 11–15, severe 16–20 and critical 21–25. Two radiologists with more than 10 years of thoracic-imaging analysis experience evaluated the severity of images in a double-blind manner. Another, more experienced, radiologist resolved any disagreement between the two radiologists determining a radiological consensus.

In addition, a qualitative assessment including the evaluation of the following CT findings, ground glass opacities (GGOs), consolidation and crazy paving, was defined according to the Fleischner Society glossary [92].

### 2.5. Statistical Analysis

Continuous data were expressed in terms of median values and range. Chi square test, Mann Whitney test and Kruskal Wallis test were used to verify differences among groups. Intraclass correlation coefficient was used to analyze the correlations and variability among quantitative measurements generated by automatic tool and radiological severity score.

Bonferroni correction was considered for multiple comparisons.

*p* value < 0.05 was considered significant for all tests.

The statistical analyses were performed using the Statistics and Machine Toolbox of MATLAB R2021b (MathWorks, Natick, MA, USA).

## 3. Results

According to the inclusion and exclusion criteria, the patient study group was composed of 13 vaccinated patients affected by the Omicron variant (Omicron V). As control groups we identified: 20 unvaccinated patients with Alpha variant (Alpha NV); 20 unvaccinated patients with Delta variant (Delta NV); 18 vaccinated patients with Delta variant (Delta V); and 20 unvaccinated patients affected by the Omicron variant (Omicron NV). Mean age and sex distribution for each group is reported in Table 1.

No differences between the groups under examination were found (*p* value > 0.05 at Chi square test) in terms of risk factors (cardiovascular diseases, diabetes, immunosuppression, chronic kidney, cardiac, pulmonary, neurologic, and liver disease, etc.).

**Figure 3:**
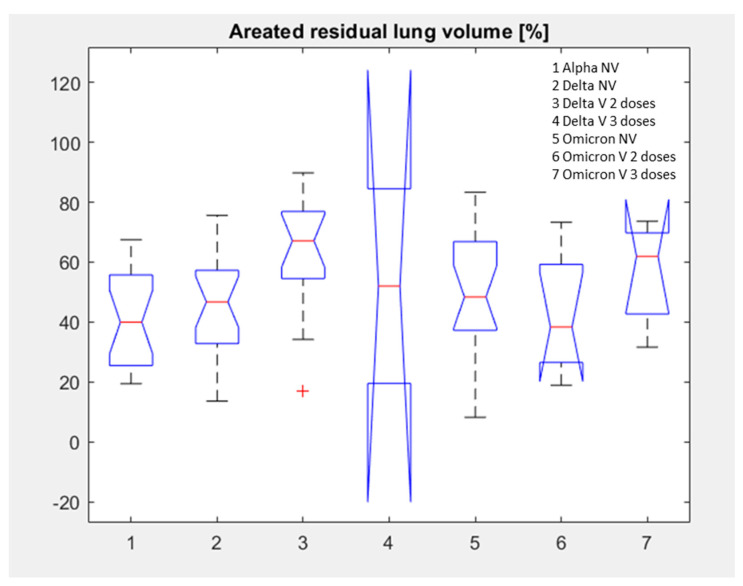
Distribution of aerated residual lung volume for each subgroup.

The patient distribution with median value of aerated residual lung volume for each subgroup is reported in Table 2 and Figure 3.

**Figure 4:**
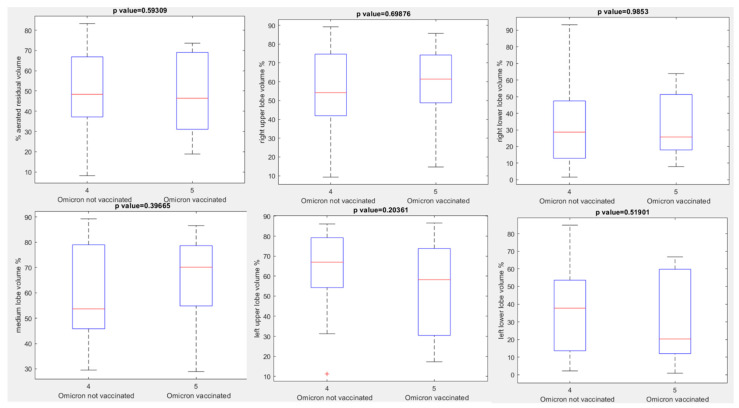
Boxplots of extracted volumes by automatic tool between vaccinated and unvaccinated patients affected by Omicron Variant of COVID-19.

No statistically significant differences were observed between unvaccinated and vaccinated patients with Omicron variant for aerated residual lung volume, right upper lobe volume, right lower lobe volume, medium lobe volume, left upper lobe volume, or left lower lobe volume in percentage values: *p* value > 0.05 with Kruskal Wallis test (see boxplots in Figure 4, Table 3).

**Figure 5:**
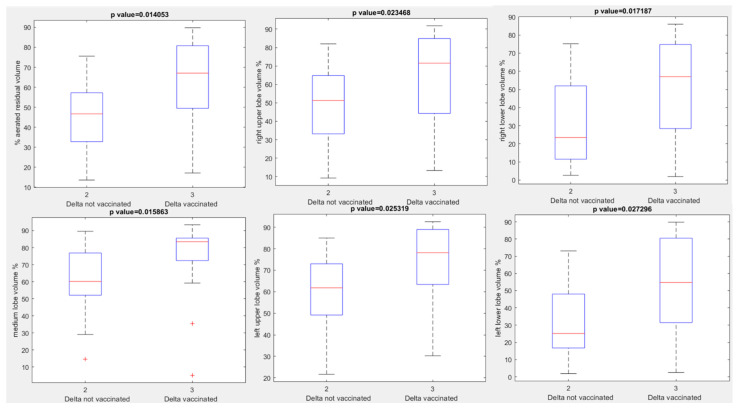
Boxplots of extracted volumes by automatic tool between vaccinated and unvaccinated patients affected by Delta Variant of COVID-19.

A different median value of aerated residual lung volume was observed in the Delta variant groups: median value of aerated residual lung volume was 46.70% in unvaccinated patients compared to 67.10% in vaccinated patients (*p* value = 0.01 with Kruskal Wallis test). In addition, in patients with Delta variant every other extracted volume by automatic tool showed a statistically significant difference between vaccinated and unvaccinated group (see boxplots in Figure 5, Table 3): *p* value at Kruskal Wallis test = 0.02, 0.02, 0.02, 0.03, 0.03, respectively, for percentage values of right upper lobe volume, right lower lobe volume, medium lobe volume, left upper lobe volume and left lower lobe volume.

**Figure 6:**
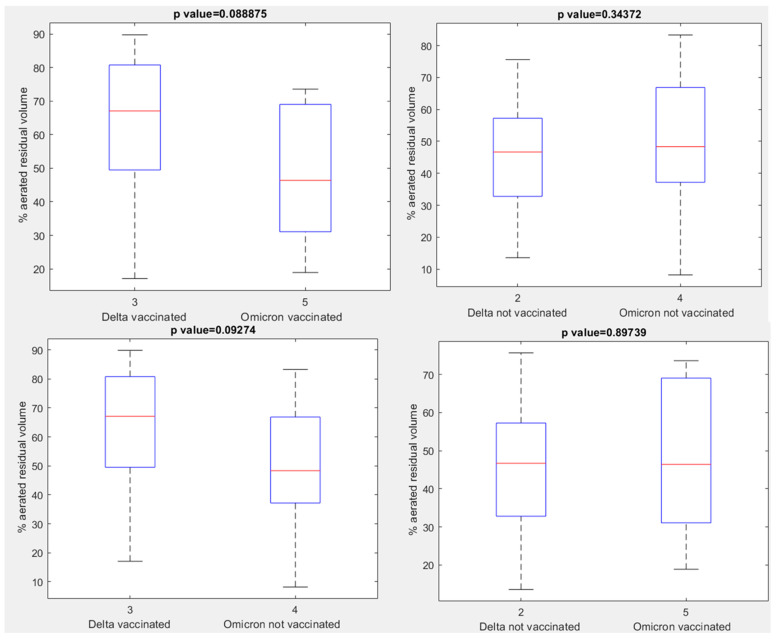
Boxplots of aerated residual volume between unvaccinated or vaccinated patients affected by Delta variant and vaccinated or un-vaccinated patients affected by Omicron variant.

**Figure 7:**
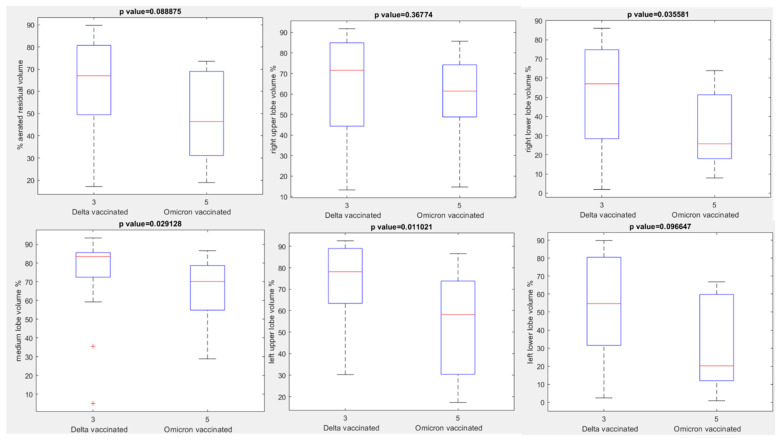
Boxplots of extracted volumes by automatic tool between vaccinated patients affected by Delta Variant of COVID-19 and vaccinated patients affected by Omicron Variant of COVID-19.

**Figure 8:**
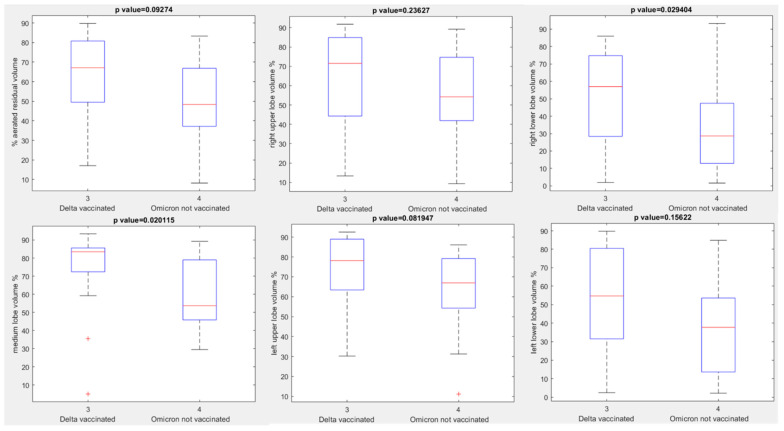
Boxplots of extracted volumes by automatic tool between vaccinated patients affected by Delta Variant of COVID-19 and unvaccinated patients affected by Omicron Variant of COVID-19.

No statistically significant differences were observed in terms of aerated residual lung volume among vaccinated or unvaccinated patients with Delta and vaccinated or unvaccinated patients with Omicron variant (*p* value > 0.05 with Kruskal Wallis test, Figure 6). The only statistically significant differences were observed between vaccinated patients with Delta variant and vaccinated patients with Omicron variant for the right upper lobe volume, medium lobe volume and left lower lobe volume with a *p* value at Kruskal Wallis test, respectively, of 0.04, 0.03 and 0.01 (Figure 7) and between vaccinated patients with Delta variant and unvaccinated patients with Omicron variant for the right upper lobe volume and medium lobe volume with a *p* value for Kruskal Wallis test, respectively, of 0.03 and 0.02 (Figure 8).

No difference was observed in terms of each extracted volumes by automatic tool (aerated residual lung volume, right upper lobe volume, right lower lobe volume, medium lobe volume, left upper lobe volume, left lower lobe volume) between unvaccinated patients with the Alpha variant versus vaccinated or unvaccinated patients with the Omicron variant (*p* value > 0.05 for Kruskal Wallis test).

**Figure 9:**
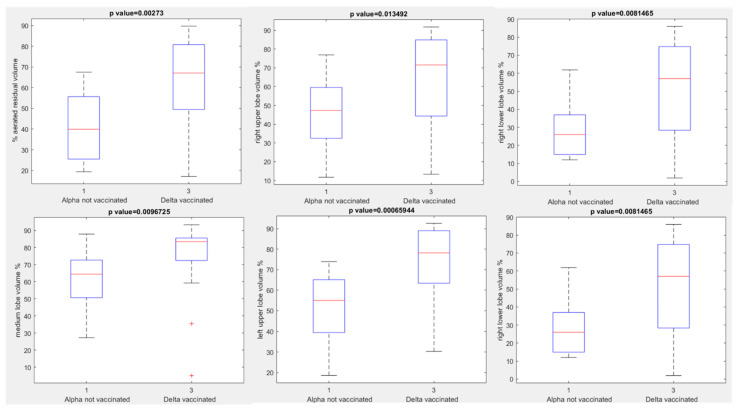
Boxplots of extracted volumes by automatic tool between unvaccinated patients affected by Alpha variant and vaccinated patients affected by Delta variant of COVID-19.

In addition, no difference was observed in terms of each extracted volume by automatic tool between unvaccinated patients with the Alpha variant versus unvaccinated patients with the Delta variant (*p* value > 0.05 for Kruskal Wallis test). Instead, statistically significant differences were observed for each extracted volume by automatic tool between unvaccinated patients affected by Alpha variant and vaccinated patients affected by Delta variant of COVID-19: *p* value for Kruskal Wallis test = 0.003, 0.01, 0.01, 0.01, 0.001, 0.01, respectively, for percentage values of aerated residual lung volume, right upper lobe volume, right lower lobe volume, medium lobe volume, left upper lobe volume and left lower lobe volume (see boxplots in Figure 9).

The highest differences were observed in median value of aerated residual lung volume (39.95% versus 67.10%) in unvaccinated patients with Alpha variant compared to vaccinated patients with Delta variant and in left upper lobe volume (55.00% versus 78.15% in unvaccinated patients with Alpha variant compared to vaccinated patients with Delta variant).

**Figure 10:**
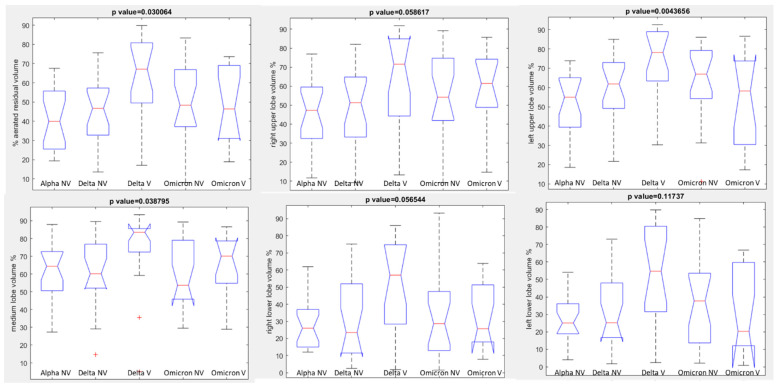
Boxplots of extracted volumes by automatic tool patients affected by Alpha, Delta or Omicron Variant of COVID-19.

Considering all groups together to assess statistically significant differences in terms of median value of extracted volumes by automatic tool, a statistically significant difference was observed in the percentage values of the aerated residual lung volume with a *p*-value of 0.03 for the Kruskal Wallis test (see boxplots in Figure 10 and Table 3) due to the highest value of aerated residual volume in vaccinated patients with Delta variant compered to every other group. 

No statistically significant difference was observed in the exitus number among groups (*p* value = 0.95 at Chi Square test).

**Figure 11:**
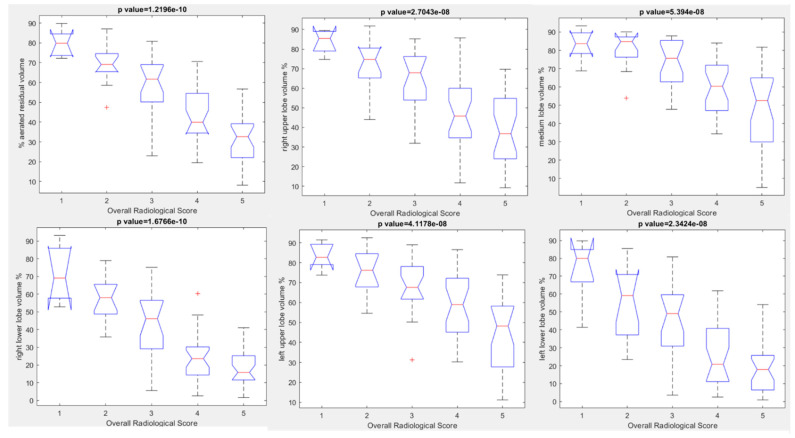
Boxplots of extracted volumes by automatic tool compared to Overall Radiological Severity score.

Good statistically significant correlations among volumes extracted by automatic tool for each lung lobe and overall radiological severity score were obtained (ICC range 0.71–0.86). Boxplots of the extracted volumes with automatic tool with respect to the overall radiological severity score are reported in Figure 11: aerated residual volume, right upper lobe volume, right lower lobe volume, medium lobe volume, left upper lobe volume, left lower lobe volume in percentage values.

Table 4 reports the median values of extracted volumes for each patient group (Alpha, Delta and Omicron group) with respect to the overall radiological severity score (from 1 to 5). No statistically significant difference was found in the overall radiological severity score for each patient group with respect to patients age (*p* value > 0.05 at Chi square test, Table 5). 

GGO was the main sign of COVID-19 lesions on CT images. CT showed multiple irregular areas of GGOs in 87 of the 91 (95.6%) patients. Consolidations were found in 70/91 (76.9%) patients and crazy paving sign in 78/91 (86.6%) patients. No statistically significant differences were observed in CT findings (GGO, consolidation or crazy paving sign) among each patient group (*p* value > 0.05 at Chi square test, Table 1).

## 4. Discussion

The debate on the efficacy of the vaccine remains, unfortunately, still open, despite the clear evidence of a reduction in the number of patients admitted to ICU [93,94]. A retrospective analysis [94], based from 465 U.S. health care facilities, showed that severe COVID-19 outcomes (i.e., respiratory failure, ICU admission, or death) were rare among adults aged ≥18 years after primary vaccination. In addition, this study showed that risk for severe COVID-19 outcome after primary vaccination was higher among persons aged ≥65 years with immunosuppression, diabetes, and chronic kidney, cardiac, pulmonary, neurologic, and liver disease [94]. However, these data were obtained among persons who acquired COVID-19 after primary vaccination during periods of pre-Delta and Delta variant predominance, so that these results should not be applicable to the risk from Omicron variant or future variants [94]. In our study we showed that in critically ill patient no difference was observed in terms of severity of disease due to pulmonary parenchymal involvement, between unvaccinated and vaccinated patients with Omicron variant, between vaccinated or unvaccinated patients with Delta and vaccinated or unvaccinated patients Omicron variant, between unvaccinated patients with the Alpha variant versus vaccinated or unvaccinated patients with the Omicron variant, or between unvaccinated patients with the Alpha variant versus unvaccinated patients with the Delta variant. Instead statistically significant differences were observed between unvaccinated patients affected by Alpha variant and vaccinated patients affected by Delta variant, and between unvaccinated patients with Delta variant versus vaccinated patients with Delta variant. The highest differences were observed between unvaccinated patients with Alpha variant compared to vaccinated patients with Delta variant. 

According to our results, no statistically significant difference was observed in the exitus number among groups. This result could be explained by the fact that the patients in the study were all admitted to ICU and for this reason in a serious condition regardless of vaccination. In addition, these results allow us to analyze several issues. Firstly, in critically ill patients the vaccine role is still controversial, and it could be explained by considering the evolution of the disease itself, where pulmonary impairment is also linked to a probable activation of the immune system [95]. Strong evidence indicates that critical illness caused by COVID-19 is qualitatively different from mild or moderate disease, even among hospitalized patients. Although most patients show mild clinical symptoms, about 20% of patients rapidly progress to severe illness characterized by atypical interstitial bilateral pneumonia, acute respiratory distress syndrome and multiorgan dysfunction. Almost 10% of these critically ill patients subsequently die. Insights into the pathogenic mechanisms underlying SARS-CoV-2 infection and COVID-19 progression are emerging and highlight the critical role of the immunological hyper-response in disease exacerbation [96,97,98]. 

Secondly, we found no difference between all groups considering pulmonary parenchymal involvement, except in the delta patient group. These data could be explained considering that the prevalence of the delta variant infection, in Italy, corresponds to the period in which the vaccination campaign was more intense, therefore without a decline in vaccine-related immunity, as suggested by emerging evidence [99,100]. A large observational study conducted using nationwide mass vaccination data in Israel showed that a third dose of the BNT162b2 mRNA COVID-19 vaccine is effective in preventing severe COVID-19-related outcomes. Compared with two doses of the vaccine administered at least 5 months before, adding a third dose was estimated to be 93% effective in preventing COVID-19-related admission to hospital, 92% in preventing severe disease, and 81% in preventing COVID-19-related death, as of 7 or more days after the third dose [101]. In our study group only a few patients had a booster dose; this data could explain to us why not only patients at risk, but also young people in apparent good health were hospitalized in intensive care, and why there were no statistically significant differences between the various risk factors in our sample.

Last but not least, is the tOmicron variant question. Our data do not allow us to establish whether the severity of the disease is linked to a decline in vaccine-related immunity or to the ineffectiveness of the vaccine against the omicron variant. At the present, there are four types of vaccines, i.e., virus vaccines, viral-vector vaccines, DNA/RNA vaccines, and protein-based vaccines [102]. Essentially, the current COVID-19 vaccines in use mainly target the S protein [103]. The 32 amino acid changes, including three small deletions and one small insertion in the spike protein, suggest that these mutations may dramatically enhance the Omicron variant’s ability to evade current vaccines [104,105,106]. Although data has suggested the potential benefit of booster mRNA vaccines for protection against Omicron [107], further studies on a larger sample are necessary.

Our quantitative analysis was obtained by Philips IntelliSpace Portal clinical application CT COPD software, designed to quantify pulmonary emphysema in patients with chronic obstructive pulmonary disease. The tool provides segmentation of the lungs and of the airway tree. Moreover, the tool helps visualize and quantify the destructive process of diffuse lung disease (e.g., emphysema), providing a guided workflow for airway analysis, reviewing and measuring airway lumen, and assessing trapped ait. Compared to others tools, this allows assessment of consolidation. In fact, during our evaluation we used also two others tools, Thoracic VCAR Software (GE Healthcare, Chicago, IL, USA) and a pneumonia module of ANKE ASG-340 CT workstation (HTS Med & Anke, Naples, Italy). However, these tools were unable to identify consolidation in all patients and, to avoid excluding patients, we reported the results obtained with a single tool. 

The present study has limitations, first of all the assessed sample size. However, we selected critically ill patients in intensive care who had a CT study for an evaluation of the objective “gravity” of the disease. The possibility of objectively grading the disease made the data robustly comparable, eliminating the variability associated with qualitative assessment [108,109,110,111,112,113,114,115,116,117,118,119,120]. Secondly is the small number of patients who had taken a booster dose, which did not allow us to assess whether the additional dose could be protective or not. Third is the selection of the control group, linked to the need to have performed a CT study, which could be responsible for bias in the results. However, we have already explained how an objective quantification of disease severity was considered crucial. Finally, since we did not know the date of the last vaccine dose for all patients, it was not possible to evaluate the severity based on the time of immunity status.

## 5. Conclusions

The debate on the efficacy of the vaccine remains still open, despite the clear evidence of a reduction in the number of patients admitted to Intensive Care Unit. In our study we showed that in critically ill patients no difference was observed in terms of severity of disease or exitus between unvaccinated and vaccinated patients. The only statistically significant differences were observed, with regard to the severity of COVID-19 pulmonary parenchymal involvement, between unvaccinated patients affected by Alpha variant and vaccinated patients affected by Delta variant, and between unvaccinated patients with Delta variant versus vaccinated patients with Delta variant.

## Figures and Tables

**Table 1:** Demographic and CT findings of Patients in the Study.

Characteristic	Alpha Variant *n* = 20	Unvaccinated Delta Variant *n* = 20	Unvaccinated Delta Variant *n* = 18	Unvaccinated Omicron Variant *n* = 20	Vaccinated Omicron Variant *n* = 13	*p* Value
**Age (y)**						
Mean	62	58	64	69	75	0.07
Range	43–78	37–83	35–87	42–88	55–94	
**Sex, no. (%) of patients**						
Male	14	17	15	13	12	0.43
Female	6	3	3	7	1	
**CT Findings**						
GGO	19	20	16	19	13	0.89
Crazy Paving	17	20	14	16	11	0.10
Consolidation	15	17	11	16	11	0.70
**Exitus**	5	5	6	4	5	0.95

Note. *p* value was evaluated for continuous variable by Mann Whitney test and by Chi square test for categorical variables.

**Table 2:** Patient distribution and median value of aerated residual lung volume for each subgroup.

		Unvaccinated	Vaccinated with 2 Doses	Vaccinated with 3 Doses	*p* Value
Patients with Alpha Variant	Number of patients	20	0	0	0.001
Patients with Delta variant		20	16	2	
Patients with Omicron		20	8	5	
Patients with Alpha Variant	Median value of (range) of Aerated residual lung volume [%]	39.95 (19.40–67.50)	-	-	0.05
Patients with Delta variant		46.7 (13.60–75.60)	67.10 (17.10–89.80)	52.00 (19.40–84.50)	
Patients with Omicron		48.35 (8.20–83.30)	38.30 (18.90–73.30)	61.9 (31.60–73.60)	

**Table 3:** Median values of extracted volumes by automatic tool patients affected by Alpha, Delta or Omicron Variant of COVID-19 grouped by vaccination or no vaccination.

	Aerated Residual Volume %	Right Upper Lobe Volume %	Right Lower Lobe Volume %	Medium Lobe Volume %	Left Upper Lobe Volume %	Left Lower Lobe Volume %
**Alpha**	39.95	47.30	26.00	64.40	55.00	25.05
Unvaccinated	39.95	47.30	26.00	64.40	55.00	25.05
**Delta**	55.25	56.2	58.35	72.9	32.75	56
Unvaccinated	46.70	39.20	51.30	60.15	23.45	46.65
Vaccinated	67.10	66.50	71.55	83.50	57.00	66.80
**Omicron**	46.4	46.8	59	68.4	26.9	50
Unvaccinated	48.35	42.2	54.2	53.65	28.65	51.65
Vaccinated	46.4	49.8	61.4	70.1	25.7	45.1
***p* value at Kruskal Wallis test**	0.03	0.06	0.06	0.04	0.004	0.12

**Table 4:** Median values of extracted volumes by automatic tool for patients affected by Alpha, Delta or Omicron Variant of COVID-19 grouped by overall radiological severity score.

	Overall Radiological SCORE	Aerated Residual Volume %	Right Upper Lobe Volume %	Right Lower Lobe Volume %	Medium Lobe Volume %	Left Upper Lobe Volume %	Left Lower Lobe Volume %
Alpha	2	57.50	65.40	48.85	70.50	59.50	26.30
	3	47.37	72.07	35.27	80.50	66.67	33.03
	4	39.51	40.20	23.73	54.84	49.61	27.15
	5	36.96	39.06	23.71	57.19	44.23	25.16
Delta	1	82.17	86.83	73.63	87.37	84.87	71.67
	2	76.06	74.02	68.02	86.20	83.06	66.08
	3	65.25	68.04	54.91	75.65	72.97	56.29
	4	43.40	47.19	21.51	63.44	61.50	20.47
	5	26.86	29.34	11.54	38.18	45.60	15.18
Omicron	1	77.73	80.83	69.03	78.43	81.53	75.93
	2	67.97	72.62	51.53	78.83	75.50	57.17
	3	47.87	55.15	25.83	65.77	60.47	29.38
	4	46.64	52.84	29.56	57.07	59.79	31.39
	5	34.00	47.12	16.17	51.25	44.08	15.88
*p* value at Kruskal Wallis test		<0.001	<0.001	<0.001	<0.001	<0.001	<0.001

**Table 5:** Overall Radiological Severity Score correlated with patients’ age for each group.

	Overall Radiological Severity Score	Alpha Variant *n* = 20	Delta Variant *n* = 38	Omicronvariant *n* = 33	*p* Value at Chi Square Test
≤65 years	≤5	0	2	3	0.55
>65 years		0	1	0	
Total		0	3	3	
≤65 years	6–10	1	2	5	0.32
>65 years		1	3	1	
Total		2	5	6	
≤65 years	11–15	0	7	4	0.11
>65 years		3	4	2	
Total		3	11	6	
≤65 years	16–20	4	3	6	0.06
>65 years		4	8	1	
Total		8	11	7	
≤65 years	21–25	3	3	8	0.25
>65 years		4	5	3	
Total		7	8	11	

## Data Availability

All data are reported in the manuscript.
